# Washing and Baking Sodas

**Published:** 1888-09

**Authors:** 


### WASHING AND BAKING SODAS.

### HOW THEY ARE MADE.

Strange as it may seem, about all of our washing and baking soda is obtained indirectly from our common table salt. Notwithstanding the fact that large quantities of these compounds occur in many parts of the earth, it has been found more profitable and more desirable to manufacture them, even though the process is a long and complicated one, rather than to merely mine and purify the materials. Among the more important localities where they are found native may be mentioned the soda lakes of Egypt and Central Africa, the borders of the Caspian and Black Seas, in California, Mexico and many parts of South America.

The crude soda coming from the different places goes under various names, as Tro Na. Natron, Urao, &c.

Another source of supply is the ashes of sea weeds and of plants growing on the coast. The sea weeds are collected by the inhabitants, dried, burned and the ashes treated with water, lixiviated, as is technically termed. The water is then boiled down or concentrated, when the soda crystalizes out. Though the yield of soda from a given quantity of sea weed is very small, it being said that it takes twenty-four tons of dried weeds to make 50 to 100 pounds of soda, still there are a great many people engaged in the business ; at one time over 20,000 people in the Orkney Islands alone followed it for a livelihood. Of course, there are a few other substances obtained at the same time which somewhat increase the income, but the soda is the main one. The soda from this source also has several names, among the more common of which are Barilla, Salsola Soda and Varec.

But the amount of soda from all of these sources sinks into insignificance when compared with that produced from common salt by the chemical processes. The most important and commonly employed of these, of which there are several, is perhaps that invented by M. Leblanc, a native of France. This process was brought before the public in 1793, the time of the French revolution, by the Comite du Salut Public, compelling all soda manufacturers to send them a full description of their methods of manufacture, giving the most minute details of every step of the operation. That of Leblanc was judged the best and has stood foremost up to the present time. The full particulars of his mode of working were published and presented to the public without giving him the slightest remuneration for his invention, and hence one of the greatest benefactors of mankind died poor. Not only does this process furnish us with the material from which to make our washing and baking soda and the principal ingredient of baking powders, but it also has given us the means for the cheap production of hard soap, a most necessary article to civilized man, and of glass.

The outline of this method of manufacture may be briefly described as follows :

In a specially constructed furnace well-warmed oil of vitrol or sulphuric acid is added in certain proportions to heated common salt. A violent action takes place and immense quantities of an exceedingly suffocating gas are given off. When the reaction has ceased the mixture is shovelled or raked into another part of the furnace, where it is heated to a much higher temperature. More of the same gas is now given off and the substance formed on the mixing of the acid and salt, chemically known as bisulphate of soda, is converted into the normal sulphate of soda, or, as it is commonly called, Glauber's Salts. This is the "salt cake" of the factory hands. For a long time the gas given off was a source of great trouble. It is extremely soluble in water forming muriatic acid, and it is from this source that we obtain the acid found in commerce. Now, at first the gas was allowed to escape directly into the atmosphere from the factory chimneys and and uniting with the watery vapor fell as a rain of muriatic acid, killing all vegetation within a long distance, and even the fish in the neighboring streams. Thus many fertile parts of the country were converted into barren wastes. This continued until a law was passed compelling the manufacturers to abate the nuisance, which was accomplished by passing the gas through "scrubbers." These are high towers filled with coke, over which water is kept constantly trickling. The water absorbs the gas and thus the difficulty is overcome. The resulting liquid (crude muriatic acid) is largely used in making bleaching powder, and is really about the only source of profit now to the soda manufacturers. We have here a very good illustration of the fact that a substance which is regarded as a waste product and thrown away, often turns out to be more valuable than the main substances ought to be obtained when its composition has been determined and a use found for it. How well do we see this same fact demonstrated in the case of the manufacture of common illuminating gas. A few years ago men were paid for carting away waste material which to-day produces several times the amount of profit that all the coke and gas together pay.

But to go on with our process; the "salt cake" is mixed with chalk or marble and coal, the mixture is put into what is known as a reverberatory furnace and fused. The sulphate of soda is converted into the carbonate of soda, and the chalk into the oxysulphide of calcium. The mixture of these two substances, which has a dark color due to the presence of some undecomposed coal, is called "black ash," while the process itself is technically known as "bailing." The "black ash" is then dissolved in water and allowed to stand so that some of the impurities and the coal can settle. This solution after careful decantation is evaporated to dryness and the resulting solid heated to redness. Soda ash or crude carbonate of soda is the result, and it is from this substance that our washing and baking sodas are directly made.

To obtain washing soda the soda ash is dissolved in hot water until the water will not dissolve any more and the solution allowed to stand until all the solid impurities have settled to the bottom. The clear liquid is then carefully drawn off into very shallow iron pans so that a large surface may be exposed to the air and the evaporation of the water go on more rapidly. The refined carbonate of soda gradually crystallizes, but forming large masses. These masses of crystals are now collected, and after the adhering liquid has been drained from them they are packed in barrels and sent into commerce as washing soda. In this condition they contain over 60 per cent. of water, hence when one buys ten pounds of washing soda he pays for six pounds of water and but four pounds of soda.

To make baking soda they dissolve the soda ash in hot water and let it stand and settle as in making washing soda, but it is further purified by filtering it through layers of black sand and bone black. The soda is then crystallized out and drained. After draining it is ground into a coarse powder and formed into cakes having holes running through them. These cakes are placed on peforated shelves in specially constructed air-tight rooms, which have a capacity of about ten tons of soda. After the room has been filled the door is closed and locked and carbonic acid gas, formed by burning coal, is forced in by means of a blower. The soda remains in this atmosphere for from three weeks to a month, at the end of which time it has absorbed enough of the gas to change it from the carbonate to the bicarbonate of soda, which latter is our baking soda. The room is now opened and the cakes taken out. Each cake is broken into halves and the fresh surfaces examined by an expert, who judges by their appearance to what grade the pieces belong. The sorting over, the cakes are put into a machine resembling a coffee mill, where they are ground to a coarse powder. This powder is conveyed to other mills, which grind it very fine. It is then packed in boxes and casks and sent into the market.

Bicarbonate of soda is very often put up in packages and labeled saleratus, but this is an adulteration or rather a fraud, as real saleratus is the bicarbonate of potash and is obtained from entirely a different source, There is, however, an easy method of distinguishing between the two. If some of the suspected substance be sprinkled pretty thickly over some loose cotton and saturated with alcohol and the alcohol set on fire, the flame will be bright yellow if the substance is baking soda, and purplish if it is real saleratus.—*Anti-Adulteration Journal.*

